# Measuring Extracellular Vesicles by Conventional Flow Cytometry: Dream or Reality?

**DOI:** 10.3390/ijms21176257

**Published:** 2020-08-29

**Authors:** Donatella Lucchetti, Alessandra Battaglia, Claudio Ricciardi-Tenore, Filomena Colella, Luigi Perelli, Ruggero De Maria, Giovanni Scambia, Alessandro Sgambato, Andrea Fattorossi

**Affiliations:** 1 Department of Translational Medicine and Surgery, Università Cattolica del Sacro Cuore, 00168 Rome, Italy; dnlucchetti@gmail.com (D.L.); c.ricciarditenore@hotmail.it (C.R.-T.); colella.filomena@gmail.com (F.C.); luigi.perelli19934@libero.it (L.P.); demariaruggero@gmail.com (R.D.M.); 2 Department of Life Science and Public Health, Università Cattolica del Sacro Cuore, 00168 Rome, Italy; alessandra.battaglia@unicatt.it; 3 Laboratory of Cytometry and Immunology, Department of Obstetrics and Gynecology, Università Cattolica del Sacro Cuore, Fondazione Policlinico Universitario A. Gemelli IRCCS, 00168 Rome, Italy; giovanni.scambia@policlinicogemelli.it (G.S.); andrea.fattorossi@guest.policlinicogemelli.it (A.F.); 4 Centro di Riferimento Oncologico della Basilicata (IRCCS-CROB), Rionero in Vulture (PZ), 85028 Potenza, Italy

**Keywords:** extracellular vesicles, exosomes, flow cytometry, immunophenotyping, swarm detection

## Abstract

Intense research is being conducted using flow cytometers available in clinically oriented laboratories to assess extracellular vesicles (EVs) surface cargo in a variety of diseases. Using EVs of various sizes purified from the HT29 human colorectal adenocarcinoma cell line, we report on the difficulty to assess small and medium sized EVs by conventional flow cytometer that combines light side scatter off a 405 nm laser with the fluorescent signal from the EVs general labels Calcein-green and Calcein-violet, and surface markers. Small sized EVs (~70 nm) immunophenotyping failed, consistent with the scarcity of monoclonal antibody binding sites, and were therefore excluded from further investigation. Medium sized EVs (~250 nm) immunophenotyping was possible but their detection was plagued by an excess of coincident particles (swarm detection) and by a high abort rate; both factors affected the measured EVs concentration. By running samples containing equal amounts of Calcein-green and Calcein-violet stained medium sized EVs, we found that swarm detection produced false double positive events, a phenomenon that was significantly reduced, but not totally eliminated, by sample dilution. Moreover, running highly diluted samples required long periods of cytometer time. Present findings raise questions about the routine applicability of conventional flow cytometers for EV analysis.

## 1. Introduction

Extracellular vesicles (EVs) are membrane-surrounded structures released in the intercellular environment and blood stream by a large variety of cells. EVs shuttle lipids, proteins, RNA, DNA, and other metabolites between cells and tissues. They diverge into two main subgroups according to their biogenesis and release mechanism: microvesicles (150–1000 nm in diameter, MVs) shed from the plasma membrane, and exosomes, which are generally smaller in size (30–150 nm in diameter, EXOs) originating from the endosome as intraluminal vesicles enclosed within multivesicular bodies [1]. EVs are central in regulating multiple physiological processes—e.g., tissue repair, stem cell maintenance and coagulation―and pathophysiological processes―e.g., cancer, neurodegenerative diseases and viral infections [2]—because of their ability to transfer biological content. Since EVs are found in accessible body fluids and express a variety of bioactive molecules of the cells of origin, intense research is being conducted to understand EVs’ potential as biomarkers for personalized medicine, and to develop relatively simple and fast methods to assess EVs in translational studies using high-throughput technologies.

Indeed, a number of techniques are potentially suited to assess individual EVs, including electron microscopy, resistive pulse sensing, nanoparticle tracking analysis, dynamic light scatter (DLS), and flow cytometry [3]. However, only the latter technique is able to combine high-throughput and adequate speed allowing EVs evaluation in translational studies and in a routine clinical setting. Several custom-constructed flow cytometers or last generation modified cell sorter, with optimized fluidics and flow cell design, have been developed to detect extremely small particles [4,5]. However, these instruments are not optimally suited for other more common applications in clinical settings, mostly cell immunophenotyping. Paradoxically, it is in the clinical setting that EVs are currently most extensively investigated by flow cytometry in a variety of pathological processes.

The current generation of commercial flow cytometers include highly complex and sensitive instruments, which are optimized to assess lymphocytes and other similar sized cells. Commercially available flow cytometers routinely measure light scatter in the forward scatter (FSC) and right angle, or side scatter (SSC), directions, and the two parameters combined provide a good foundation to begin cell population analysis. To identify a particle, the scattered light must exceed the triggering threshold, which must be set to exclude the optical and electronic noise. This is easily accomplished when analyzing micrometer-sized particles, such as cells and the largest EVs, e.g., apoptotic bodies. Conversely, smaller EVs generate scatter signals that may be extremely low and fall within the range of the optical and electronic noise; because the intensity of the scattered light attenuates exponentially with size (the sixth power of particle size) [6], these EVs remain hidden in the background.

In the cytometry of EVs with conventional flow cytometers, FSC is generally less used than SSC, as only particles with a diameter larger than the typical 488 nm wavelength excitation provided by the standard blue laser preferentially scatter (in fact diffract) light in the “forward” direction. SSC is better suited to identify particles with diameters smaller than the wavelength of the incident laser light, because SSC is a measure of mostly refracted and reflected light. However, SSC signal intensity also depends on the ratio between the particle size and the wavelength of the incident laser light. To improve resolution, conventional flow cytometers have been developed to measure the SSC off the violet laser (hereafter referred to as VSSC) instead of blue laser SSC (hereafter referred to as BSSC), as the 405 nm wavelength of the violet laser compared with the 488 nm wavelength of the blue laser is closer to EVs size [7].

In the present study, we explored the feasibility to identify small/medium sized EVs (size range 70 to 300 nm) by a conventional flow cytometer, equipped with blue and violet laser excitation sources, designed to be used in clinical setting for a wide range of applications (CytoFLEX S, Beckman Coulter, Milano, Italy) [8].

We demonstrate that immunophenotyping of small sized EVs (in the 100 nm size range) is in fact not feasible, not even when the staining involves very abundant surface molecules, showing that conventional flow cytometry is inadequate for these EVs assessment. Additionally, we show that the flow cytometry analysis of medium sized EVs (around 250 nm) is plagued by an excess of coincident particles (swarm detection)―a limitation that must be considered when formulating schemes for EVs studies using conventional flow cytometers.

## 2. Results

### 2.1. EVs Generation and Flow Cytometer Set Up

EVs were obtained from the HT29 (human colorectal adenocarcinoma) cell line. EVs isolation and characterization are detailed in [9] and Supplementary Information) and shown in Figure S1. The window of analysis was determined by VSSC and fluorescence parameters using fluorescent Megamix-Plus FSC polystyrene microbeads (Figure S2). The superiority of VSSC over BSSC in terms of resolution is shown in Figure S2. During apoptotic cell death, apoptotic exosome-like vesicles similar in size and in certain surface marker expression—e.g., CD63—are released in culture supernatant [10], so we collected supernatant for EVs purification only when cell culture viability exceeded 90% by trypan blue exclusion.

### 2.2. Identification of Small and Medium Sized EVs by VSSC and Calceins

In the cytometry of EVs, fluorescence is less affected by background noise than light scatter. Thus, the poor resolution of dim light scatter signals generated by EVs can be improved by adding a fluorescent label as parameter. Among several available fluorescent labels potentially useful for EVs staining [11], we chose Calceins (Calcein-green and Calcein-violet) [12]. Calcein probes are best suited to avoid possible interferences related to staining of cell membrane fragments and non-intact EVs following ultracentrifugation procedures [13], because they require hydrolysis by intracellular esterases to become fluorescent, and, therefore, identify only metabolically active, intact vesicles, which can transform the non-fluorescent dye into the fluorescent form [12]. Moreover, these probes have little to no spectral overlap with each other and with the other fluorochromes we used, minimizing compensation and spreading error [14].

**Figure 1:**
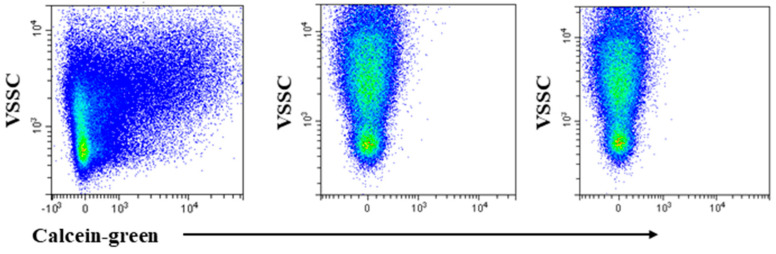
Calcein stains intact extracellular vesicles (EVs). Violet side scatter (VSSC)/Calcein-green fluorescence profile of EVs incubated with Calcein-green at 37 °C (left and right panels) and 4 °C (middle panel). The low temperature prevented the non-fluorescent Calcein from being converted into the green fluorescent form inside EVs, indicating that free dye does not contribute to the observed fluorescence pattern. Triton-X-100 treatment abrogates Calcein fluorescence at 37 °C (right panel). Using Calcein-violet produced identical results.

Unless otherwise stated, we adopted 1.5 µg/mL of EVs (by Bradford assay) in a solution with either Calcein-green or Calcein-violet at final concentration of 1 µM in filtered PBS. Most experiments were performed using Calcein-green; thus, Calcein will refer to Calcein-green, unless otherwise stated (Figure 1).

To confirm previous observation [12] that Calceins only stain intact EVs, an EVs sample was incubated with the dyes at 4 °C, as this temperature inhibits internal esterase activity and, therefore, prevents the non-fluorescent Calcein form conversion to the fluorescent form. Data in the left and middle panel of Figure 1 were generated by staining samples with Calcein at 37 °C and 4 °C, respectively. EVs remained non-fluorescent when incubated at low temperature. Subsequently, as any disruption of plasma membrane, obtained with detergents, leads to leakage of the dye from particle, we treated Calcein-stained EVs with Triton-X-100. In accordance with a previous report [12], positive events disappeared following Triton-X-100 treatment, thus excluding the contribution of free esterases to Calcein signal (Figure 1, right panel).

Calcein staining was dim to moderate, yet the distinction between positive and negative events, remained discernible by visual inspection (Figure 1, left panel). These findings seem at odds with those of De Rond et al. [15] that deemed Calcein as scarcely sensitive. We can only conjecture as to why our findings differ from previous results because the lack of a detailed description of the gating strategy and particle size in the De Rond study [15] make a comparison difficult. 

After demonstrating that the inclusion of Calcein as a second parameter was viable to identify EVs, we applied this procedure to purified medium and small sized EVs

**Figure 2:**
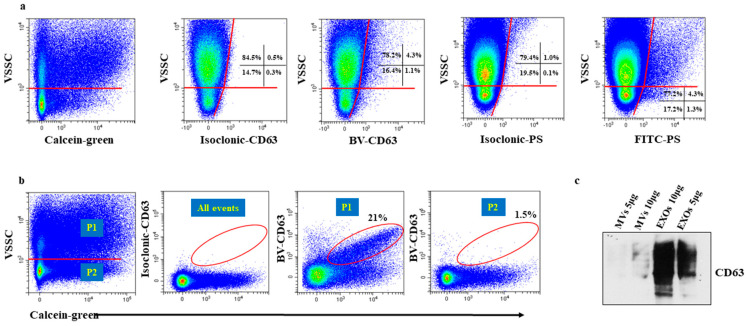
Immunostaining of purified medium and small sized EVs, (**a**) and (**b**), respectively. Both in (**a**) and (**b**), two populations can be visually discerned based on the intensity of the VSSC signal (horizontal red line). (**a**) Left panel, in this representative experiment the percentage of events with high VSSC signal intensity was 84%. Second panel from left, to demonstrate specificity of staining and set the quad markers, the binding of brilliant violet-conjugated CD63 (BV-CD63) moAb was blocked by pre-incubation with unlabelled CD63 moAb prior to staining with the BV-CD63 moAb (isoclonic control). Third panel from left, only a marginal amount of events reacted with the BV-CD63 moAb, consistent with the non-exosomes (EXOs) nature of these particles. Fourth panel from left, to demonstrate specificity of staining and set the quad markers, the binding of FITC (Fluorescein isothiocyanate)-conjugated anti- Phosphatidylserine (PS) moAb was blocked by pre-incubation with unlabelled anti-PS moAb prior to staining with the anti-PS moAb (isoclonic control). Right panel, a detectable proportion of medium sized EVs expressed PS demonstrating that immunophenotyping EVs of that size is feasible. (**b**) Left panel, in this representative experiment, the percentage of events with high VSSC (P1) and low (P2) signal intensity was 43% and 57%, respectively. Middle panel, isoclonic control. Right and far right panels, BV-CD63 staining in P1 and P2 populations, respectively. BV-CD63 moAb reacted only with events in the P1 region, consistent with the EXOs nature of these particles. Values in regions show percentages of positive events. We addressed the issue of possible BV-CD63 fluorescence quenching by Calcein because of the spectral characteristics of BV fluorochrome and run side by side small sized EVs stained with only BV-CD63 or double stained with BV-CD63 and Calcein. (**c**) Representative western bot assay showing the preferential expression of CD63 by small sized EVs. Red lines = analysis gates; red circles = EVs population analyzed.

The left panel of Figure 2a shows that, in purified medium sized EVs preparations, most Calcein positive events generated a VSSC signal that roughly corresponded to that of the 100 nm microbeads (Figure S2a, left panel). Thus, owing to the medium sized EVs distribution of 210 ± 49 nm (Figure S1), it can be inferred that these EVs generated a scatter signal with intensity comparable to that of the 100 nm polystyrene beads. This is well in line with the notion that the inner refractive index of EVs is less than 1.4, which is considerably lower than that of polymer beads (generally ~1.6) [16].

A Calcein positive particle population (~15% in six independent experiments) with a clearly lower VSSC signal intensity was also visible (Figure 2a left panel). We hypothesized that these events reflected contaminating small sized EVs deriving from imperfect ultracentrifugation procedures, which remained undetected by the DLS analysis because of their low frequency. EVs are defined according to their modality of generation, rather than size. However, microvesicles (MVs) are generally larger than exosomes (EXOs), thus, we inferred that small sized EVs samples were mostly EXOs and medium sized EVs samples were mostly MVs. It is known that CD63 and CD9, two tetraspanins, are specially enriched in EXOs membranes and scarcely expressed by MVs [17,18], and Western blot analysis of small and medium sized EVs samples showed that only the former exhibited a strong reactivity for CD63 (Figure 2c). Thus, Calcein-stained medium sized EVs samples were incubated with monoclonal antibodies (moAbs) to CD63 and CD9, to determine the origin of particles with lower VSSC signal intensity in medium sized EVs samples. Virtually no particle stained positive for CD63 (Figure 2a, second panel from left). Although these findings suggests a non-EXOs nature of these particles, this issue will be discussed again below. The moAb to CD63 marginally stained particles in the high VSSC region―a finding consistent with the low CD63 expression by MVs [17,18]. 

MVs stained positive for anti-Phosphatidylserine (PS) antibody, demonstrating that the immunophenotyping of medium sized EVs is possible [19] (right panel, Figure 2).

Running purified small sized EVs, we observed that a large proportion of Calcein positive particles was located in the high VSSC region (Figure 2b, left panel), which is a region that corresponds to that of the ~250 nm sized particles (Figure 2a, left panel) and therefore, visibly exceeds the size of these EVs (68 ± 7 nm by DLS, Figure S1). We reasoned that these particles could be aggregates, possibly generated by high-speed centrifugation [20]. In the cytometry of cells, the aggregate issue is usually addressed by checking the height, area, and width of the FSC and/or SSC pulse. In the cytometry of EVs, the exceedingly small pulse makes this conventional doublet discrimination impossible, because the magnitude of difference in any pulse parameter between an aggregate and a single event is small, and requires the resolution of a linear scale, which is unfeasible, as scatter parameters require a logarithmic scale. To determine the origin of larger particles in small sized EVs samples, we incubated small sized EVs samples with the moAb to CD63 and CD9, and analyszd the staining in relationship with VSSC signal intensity. Figure 2b, third panel from left, shows that CD63 staining was detectable only in particles with high VSSC signal intensity (Figure 2b, right panel), particles with low VSSC signal intensity remained non-fluorescent. These data supports our hypothesis that particles with high VSSC signal intensity were aggregates. However, it remains unclear why such a large proportion of aggregates remained undetected by DLS analysis. Although it is held that DLS fails to detect small proportions of aggregates in otherwise homogeneous EVs preparations [21], the proportion of aggregates we observed in our experiments is too large to remain undetected. We hypothesize that the cytometer preferentially detected aggregates due to their larger size, while most non-aggregated particles are below the VSSC threshold, and are simply not “seen” by the instrument. An alternative, not mutually exclusive explanation, is that the larger particles are coincident events (swarm detection), an issue that is addressed below.

### 2.3. Particles in the Low VSSC Signal Region Do Not Stain for Tetraspanins Because of an Insufficient Number of Binding Sites

**Figure 3:**
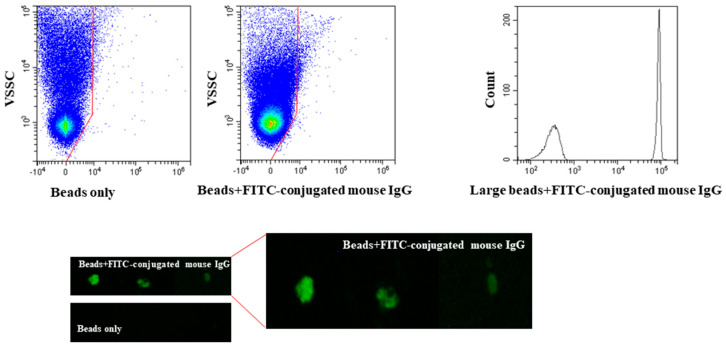
Fifty nm-sized anti-mouse IgG microbeads do not bind enough FITC-conjugated mouse IgG to generate a measurable fluorescence signal. Anti-mouse IgG microbeads background fluorescence (**top left panel**) and anti-mouse IgG microbeads coated with FITC-conjugated mouse IgG fluorescence (**top middle panel**). The same amount of FITC-conjugated mouse IgG generates a brilliant fluorescence signal when incubated with larger-sized (3 μm) microbeads coated with anti-mouse IgG (**top right panel)**. Fluorescence microscopy analysis of anti-mouse IgG microbeads carrying FITC-conjugated mouse IgG (**lower panel**). Red lines in top panel = analysis gates.

As outlined above, findings in Figure 2 indicated that the nature of the events in the low VSSC region in both the small and medium sized EVs samples could not be assessed by staining for the typical small sized EVs (mostly EXOs) markers. The analyses of medium sized EVs indicated that ~250 nm EVs were positioned in the high VSSC region (Figure 2a), and the intensity of the scattered light attenuates exponentially with size [21], so we infer that the EVs size in the low VSSC region should be considerably lower, possibly around 70 nm, in line with DLS data. Studies showed that the number of antigenic sites, which can react with a fluorochrome-labelled moAb on the surface of an EV in this size range, is around 10/15 epitopes, which is too scarce to generate a fluorescence signal detectable by a commercial flow cytometer [22,23,24]. Thus, we hypothesized that the absence of EVs immunolabeling in the low VSSC region reflected insufficient target epitopes. To verify this hypothesis, we mimicked fluorescence generated by the anti-tetraspanins fluorochrome-conjugated moAbs using FITC-conjugated mouse IgG (Immunoglobulin G) bound to 50 nm–sized anti-mouse IgG microbeads. The experiments were performed at various microbeads/FITC-conjugated mouse IgG ratios. No fluorescence signal was ever detected in the low VSSC region (Figure 3 left and middle panel). To insure that the amount of FITC-conjugated mouse IgG was enough to produce a detectable fluorescence signal in the presence of particles carrying a suitable number of binding sites, the lowest amount of FITC-conjugated mouse IgG, which was incubated with the 50 nm microbeads, was incubated with larger (3μm) microbeads coated with anti-mouse IgG. In this condition, FITC-conjugated mouse IgG generated a brilliant fluorescence signal (Figure 3 right panel). We also ascertained by fluorescence microscopy that FITC-conjugated mouse IgG made the 50 nm–sized anti-mouse IgG microbeads fluorescent (Figure 3, lower panels).

Thus, assessing the surface cargo of small sized EVs is a difficult task using conventional flow cytometers, because the fluorescence signal may remain hidden in the background.

### 2.4. Swarming and Abort Rate Affect EVs Detection

Unlike cells, EVs are not prone to align and traverse the laser path in a single row and separated from each other because of their small size. Therefore, swarm detection is the most common cause for spurious or artifactual results in the cytometry of EVs. As outlined above, we found that immunophenotyping of small sized EVs (in the size range of most EXOs) was essentially impossible. In a clinical setting, immunophenotyping is the most common approach to identify the EVs populations of interest in bodily fluids. Therefore, small sized EVs were excluded from further investigation, and we focused on medium sized EVs to investigate how swarm detection affected their analysis.

Studies suggested that the occurrence of swarming detection can be verified by serial sample dilution experiments [24,25,26]; upon dilution, the number of particles simultaneously traversing the laser beam should progressively reduce to a single particle, and the number of counted events should then decrease linearly in response to further dilution. Concomitantly, the intensity of fluorescence signal generated by the many particles coincidentally traversing the laser beam should progressively decline upon dilution and finally remain stable, which is consistent with analysis of single particles.

Thus, we quantitated changes in measured medium sized EVs concentrations and fluorescence intensity at various sample dilutions. Concentration (events/l) was calculated using the volumetric measurement featured in the CytoFLEX S cytometer (CytoFLEX S, Beckman Coulter, Milano, Italy).

**Figure 4:**
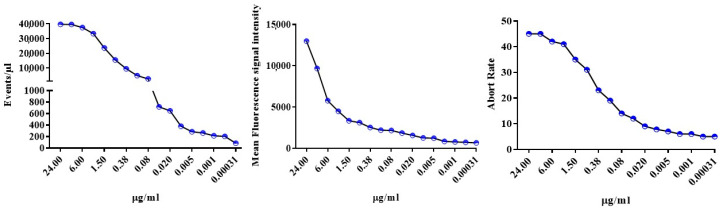
Four-fold dilutions experiments comparing changes in estimated Calcein-stained MVs concentration, fluorescence intensity and abort rate yielded not univocal results. **Left panel**, the measured MVs concentration decreased in proportion to the dilution suggesting single particle detection. **Middle** and **right panel**, fluorescence intensities and the abort rate, respectively, almost levelled only at the last four dilutions as it is the case for single particle detection. Data are from one experiment representative for the other four experiments conducted.

A relation between particle concentrations measured by the cytometer and sample dilutions was noted, which was more evident at higher dilutions, leading to hypothesize the occurrence of detection as single event (Figure 4, left panel). However, the fluorescence signal intensity never levelled completely (Figure 4 center panel). Collectively, these findings suggest that dilution reduces but does not fully prevent swarm detection.

As a result of swarming, the flow cytometry of EVs is plagued by a high abort rate; the electronics is still processing the previous pulse and aborts the new event that, consequently, is lost to analysis. Thus, we hypothesized that if EVs were detected as single events as a consequence of dilution, the abort rate would be concomitantly reduced. Figure 4, right panel, shows that the abort rate declined upon dilution, a finding that concurs with data shown in the left and center panels, supporting the progressive decrease of swarm detection. Of note, minimizing swarm detection required a progressive increase of flow cytometry time; at the highest dilution a single run required ~40 min (including washing to prevent carry-over) to collect at least 5000 Calcein positive events. 

**Figure 5:**
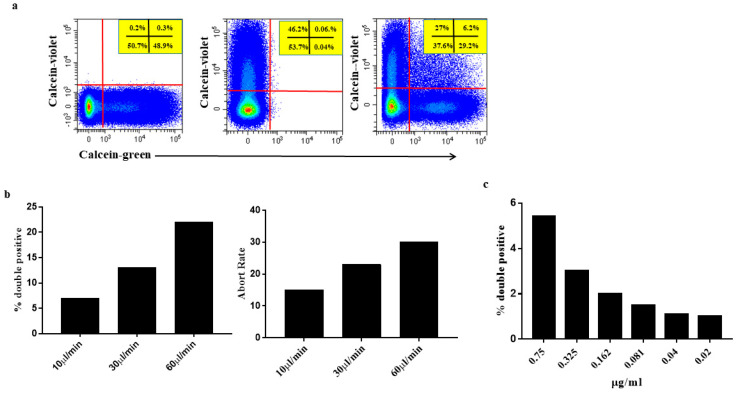
Two aliquots of the same medium sized EVs preparation were separately labelled, one with Calcein-green and the other with Calcein-violet, kept refrigerated and mixed at 1:1 ratio just before analysis. (**a**) Flow cytometry profile (green and violet fluorescence) of medium sized EVs marked individually with Calcein-green (left plot) and Calcein-violet (middle plot). The two Calceins stained medium sized EVs with comparable efficiency and the fluorescence signal of each dye did not interfere with the other. In the mixture (right plot), the two dyes identified single stained particles (either Calcein-green or Calcein-violet) and double-stained particles. Double-stained particles are visible in the upper right quadstat gate. These particles are indicative of coincident events, as there should not be double positive events when the two markers in comparison (Calcein-green and Calcein-violet) are not present on the same event. Samples were run at 10 μL/min. Values in panels show percentages of positive events. (**b**) Same sample as in (**a**), but run at the different flow rates featured in the cytometer. The frequency of double stained events (left panel) increased in step with the flow rate indicating that several particles were simultaneously traversing the laser beam as a consequence of the enlarged size of sample fluid stream. Consistent with the presence of several particles in the laser beam illuminated simultaneously, the abort ratio also increased (right panel) in step with the flow rate. Data are from one experiment representative for the other three experiments conducted. (**c**) The mixture of Calcein-green and Calcein-violet medium sized EVs were diluted to further explore the effect of high dilution on swarm recognition. Samples were run at 10 μL/min. The percentage of double positive events declined with dilution and close to zero at the lowest concentrations. Red lines = analysis gates.

Subsequently, we approached the issue of swarm detection using a model that included Calcein-green MVs mixed with Calcein-violet medium sized EVs (final concentration in the tube 0.325 μg/mL each) at a 1:1 ratio just before the flow cytometry run. Under these experimental conditions, any double-stained event undoubtedly denotes more than one EVs coincidentally traversing the laser beam. Data in Figure 5a demonstrate the presence of a sizeable number of double positive events in samples containing Calcein-green and Calcein-violet medium sized EVs, consistent with swarm detection. Consistent with the concomitant presence of several particles in the flow chamber, the VSCC signal of Calcein-green/Calcein-violet double positive EVs was higher than that of either Calcein-green or Calcein-violet single positive EVs. 

To provide further experimental evidence of swarm detection and its impact, medium sized EVs samples, containing Calcein-green medium sized EVs mixed with Calcein-violet medium sized EVs, were run at higher flow rates to increase the sample fluid stream size and to boost coincidences. Thus, the samples were run at 10 μL/min―the lowest flow rate allowed by the instrument―30 μL/min and 60 μL/min. Consistent with a progressive increase of particles, simultaneously illuminated by the laser beam, the percentage of the double stained population increased in step with the flow rate (Figure 5b left panel) [23,24,25]. Additionally, the abort rate was enhanced, which further indicated the concomitant presence of several particles simultaneously traversing the laser beam, which were too close together to be processed as single events (Figure 5b right panel). Interestingly, swarm detection and the concomitant high abort rate reduced the number of particles detected. In the experiment depicted in Figure 5, the number of particles/μL was 25.2 × 10^3^, 17.3 × 10^3^ and 10.6 × 10^3^ at 10 μL/min, 30 μL/min and 60 μL/min, respectively.

After demonstrating swarm detection impact on EVs analysis, we used the Calcein-green/Calcein-violet model, to test again how dilution may reduce coincidences contributing to overcome the problem. To this end, the Calcein-green/Calcein-violet medium sized EVs mixture was progressively diluted yielding a final concentration of 0.04 μg/tube, and the samples were run at the lowest flow rate allowed by the instrument. The proportion of double positive events declined with dilution and approximated to zero (Figure 5c), comforting data obtained in the dilution experiments carried out using Calcein-green stained medium sized EVs (Figure 4). Confirming the preceding experiments (Figure 4), minimizing double positive events entailed a long flow cytometer time (≥ 40 min).

## 3. Discussion 

In the era of precision medicine, considerable interest using flow cytometry exists to assess both quantitative and phenotypic EVs characteristics in patients suffering from a variety of diseases in the hope to reveal clinically relevant EVs subpopulations. Moreover, the presence of EVs in readily accessible sources e.g., blood, urine, and cerebrospinal fluid, makes EV flow cytometric characterization particularly appealing to monitor diseases in longitudinal studies. Unfortunately, EVs size, which is well below that of whole cells, pushes flow cytometry to the edge of its lower reliability limits.

Within recent years, several dedicated flow cytometric approaches have been developed and refined for single EVs counting and for studying EVs heterogeneity in terms of surface markers [4]. Such methods generally demand extensive operator expertise for sample preparation and, most importantly, require dedicated instrumentation for sample acquisition and data analysis, which is unfeasible for most clinically oriented flow cytometry facilities using standard nonspecialized instruments.

In the present study, we investigated in depth whether small/medium-sized EVs (50 to 250 nm size range) can be detected by a conventional flow cytometer (CytoFLEX S, Beckman Coulter) designed primarily for common applications, mostly cell immunophenotyping. Using calibrated microbeads, we were able to show that the VSSC, i.e., the SSC signal off the 405 nm laser, is much more effective than the BSSC signal, i.e., the SSC signal off the 488 nm laser in detecting EVs, as already reported [7]. However, even the VSSC signal fells short to discriminate EVs over the background generated by particulate, which survived extensive buffer filtering, and by electronic noise of the system. In fact, EVs recognition was only possible by the concomitant assessment of VSSC and the fluorescence signal produced by the general fluorescent Calcein dyes.

Perhaps the most important application of flow cytometry in the EVs study is the purported ability to evaluate their surface cargo by staining with fluorochrome-conjugated monoclonal antibodies. We found that immunophenotyping of small sized EVs, i.e., below the 100 nm range, in our experience, was impossible. Our data show that the immunofluorescent signal generated by EVs of that size becomes measurable only when generated by multiple particles that traverse the laser beam at the same time and emit enough fluorescence to be detected, whereas single particles remain underneath the fluorescence sensitivity. Notably, these conclusions were drawn by staining for markers expressed at the highest possible level on the surface of the particle, emphasizing that they will extend to all the less expressed surface markers. While we cannot exclude that effective staining may depend on type/source of EVs and the markers being evaluated, the present findings cast doubts on the view that conventional flow cytometer is a convenient tool to explore such small sized EVs. This view is in line with the early literature indicating that a single EV of that size carries around 10 epitopes; one would not expect this particle to bind enough fluorescent moAb molecules to be detected by conventional flow cytometers [22,23]. Thus, great caution must be exerted when interpreting immunophenotyping data on small sized EVs. Additional uncertainties exist performing small EVs immunophenotyping; early work showed that the attachment of large labels to antibody molecules resulted in reduced antibody binding even to surface antigens of whole cells [27]. Notably, some of the commonly used fluorophores (PE—240 kDa, PE-Cy7—255 kDa) exceed the antibody size itself and attenuation by bulky fluorophores in multicolor flow cytometry has been reported [28]. 

The conclusion that assessing surface cargo of EVs in the EXOs size range (50–70 nm) by conventional flow cytometry is questionable seems to be in contrast with some earlier data, in which anti-CD63 moAb reportedly stained EXOs [29]. Comparisons are difficult because of several factors that might have affected the results, such as the modality to set the boundary between negative and positive events, the use of an isotype rather than of a more reliable isoclonic control, and the type of instrument. However, differences in the capabilities of the Apogee A 50 dedicated cytometer used in that study [24], and the CytoFLEX S conventional cytometer in our study, to resolve artificial particles of ~100 nm size, do not seem to be relevant; the Apogee A 50 depicts large angle light scatter (LALS) compared with fluorescence profiles of FITC-labelled latex beads included in our study, which closely resemble plots depicting VSSC/FITC-labelled beads. Moreover, a comparison of our results with those by Van der Pol et al. [24], suggests that the sensitivity of the scatter signals of the Apogee A 50 dedicated cytometer and the CytoFLEX S conventional cytometer is similar, as the Apogee A 50 dedicated cytometer was able to detect single 102 nm polystyrene beads [30], as we did with the CytoFLEX S. However, it should be emphasized that our conclusions, which are based on 50 nm beads as a model for small particles immunophenotyping, cannot be generalized, as different fluorescent dyes and/or different excitation sources and/or different detection devices might provide different results. This issue, as well as other related questions, will be addressed in future studies. In this scenario, in a recent paper, the CytoFLEX was reported to detect EVs as small as 60 nm, triggering with VSSC and immunophenotyping [8]. While it is possible that the surface antigen density on the EVs used in that study was much higher than that of the EVs we tested here, it should be noted that the swarming issue was not addressed, so that it cannot be excluded that multiple small sized EVs coincidentally traversing the laser spot may have contributed to the detected fluorescence signal.

Immunophenotyping was possible in larger EVs, in the 250 nm size range. However, their assessment was plagued by swarming―a finding in line with several earlier publications [24,25,26]. We also confirmed that swarming could be minimized by extensive sample dilution [24,25,26]. Unfortunately, in translational studies, particle concentration in a given sample is most often unknown, and quite hard to predict. Thus, one should measure each given sample at various dilutions, which is unfeasible in clinically oriented laboratories. Moreover, minimizing coincidences by running high diluted samples comes at the cost of very long cytometry times (up to more than half an hour in our experience) to collect enough events of interest―a constraint hardly compatible with applications in clinical research. Notably, a (unknown) portion of double stained EVs reported in immunophenotyping studies may in fact represent coincident events, because overlooked swarm detection injects ‘polluted’ events into the cytometry data [22].

In conclusion, our study emphasizes the inherent difficulties in investigating the surface cargo of small sized EVs (in the EXOs size range ~50–70 nm). Conventional flow cytometry does detect surface cargo of relatively large sized EVs (in the MVs size range ~200–300 nm). However, we must be aware of the intrinsic limitations due to swarm detection.

A limitation of the present study is that data were generated using CytoFLEX S as example of a commercially available last generation flow cytometer designed for a wide spectrum of applications, which does not necessitate labor intensive manual hardware adjustments and calibrations. We do not have direct knowledge of the performance of other commercial instruments offering the same features to assess EVs, and we are not aware of side-by-side comparative tests performed using the same EVs preparation. This latter point is quite an important one, as knowing what size and level of fluorescence a given platform is able to detect will allow for comparisons amongst different instruments, and even of the same producer, which is an important issue for standardization, and, therefore, reproducibility. We are now designing studies to allow the conversion of scattering intensity in arbitrary units to diameter distribution and of fluorescence intensity to MESF (Molecules of Equivalent Soluble Fluorochrome) to better define the detection limits of each instrument [31].

## 4. Methods

### 4.1. Reagents

Calcein-AM green (referred to as Calcein-green) and Calcein-AM violet (referred to as Calcein-violet) were purchased from Life Technologies. Both dyes were solubilized in DMSO to produce a 100x stock solution (1 mM). The nonionic detergent Triton X-100 was purchased from Thermofisher. Mouse anti-human brilliant violet-conjugated CD63 (BV-CD63, clone H5C6) and mouse anti-human R-phycoerythrin (PE)-conjugated CD9 (PE-CD9, clone HI9a) monoclonal antibodies (moAbs), as well as unconjugated identical (isoclonic control) CD63 and CD9 moAbs were purchased from BioLegend, Inc. Fluorochrome-conjugated moAbs were centrifuged before using to minimize the antibody aggregates commonly found in commercial preparations. Fifty nm sized microbeads carrying goat anti-mouse IgG (H+L) F (ab’)2 fragments were purchased from Miltenyi Biotec (referred to as anti-mouse IgG microbeads). These beads are made of very dense material to be paramagnetic, a feature that implies a refraction index at least comparable to that of polystyrene beads. The VSSC threshold we used in all experiments included events producing a signal one and half decade lower than that generated by the smallest polystyrene beads (100 nm) included in the Megamix-Plus FSC mix (Supplementary Figure S2a and Supplementary Information), so we speculated that the Miltenyi 50 nm beads might become visible if made fluorescent. FITC Mouse IgG1, κ Isotype Control (clone MOPC-21) was purchased from BD Biosciences. Megamix-Plus FSC consisting in 100 nm, 300 nm, 500 nm, and 900 nm microbeads were purchased from BioCytex. Rainbow Calibration Particles consisting in a series of beads with eight different predefined levels of fluorescence intensity, including nonfluorescent beads, were purchased from BD Biosciences. VersaComp 3μm beads were purchased from Beckman Coulter.

### 4.2. EVs Staining by Calcein-Green and Calcein-Violet, and Anti-Tetraspanins moAbs

Calcein-green and Calcein-violet are essentially colorless and non-fluorescent until hydrolyzed. Once inside the EVs, the lipophilic blocking groups are cleaved by nonspecific esterases. As a result, Calceins are trapped inside the EV and emit a green or a violet fluorescence signal (emission max 516 nm and 450 nm, respectively) following excitation with a blue (488 nm) or violet (405 nm) laser. After some preliminary experiments, the incubation time was set at 20 min at 37 °C, final concentration 1 μM All staining steps were carried out in filtered PBS.

In immunophenotyping experiments, EVs, which had been previously loaded with Calcein, were incubated with moAbs to CD63, CD9, Anti-Phosphatidylserine for 30 min at room temperature (RT). All moAbs were used at concentration of 1.25 μg/mL^−1^. All moAbs were used at 1:20 final dilution. To assess moAb non-specific binding, Calcein-loaded MVs and EXOs were incubated with a five-fold excess of identical unlabelled moAb (isoclonic control) before being reacted with the fluorochrome-conjugated moAb. Under these conditions, all specific binding sites of the fluorochrome-conjugated moAb are blocked by the large excess of unconjugated antibody and non-specific staining can be measured. Summary of reagents used for flow cytometry experiment are shown in Table 1.

To avoid false positive events, moAbs were centrifuged using 0.22 μm centrifugal filter tubes and a fixed angle single speed centrifuge (~750× *g*) at RT for 2 min. The flow through was collected and used for immunophenotyping experiments. In each staining session, the moAbs were run in filtered PBS alone to check signal from antibody aggregates that survived centrifugation.

### 4.3. Microbead Experiments

The 50 nm anti-mouse IgG microbeads served to mimic the low amount of binding sites available on an EXO-sized particle. These microbeads are designed to bind the largest possible number of moAb molecules per surface unit. The microbeads (5 to 10 μL directly from original bottle) were incubated with FITC-conjugated mouse IgG (final dilution 1:20 in filtered PBS) for 30′ at 4 °C in rotation. For comparison, the same amount of FITC-conjugated mouse IgG was incubated with 5 μL of larger sized beads (VersaComp 3 μm beads, Beckman Coulter, CytoFLEX S, Beckman Coulter, Milano, Italy), coated with anti-mouse IgG.

### 4.4. Flow Cytometry

EVs were analysed using a CytoFLEX S instrument equipped with blue (488 nm) and violet laser (405 nm) excitation sources. The instrument is able to collect SSC off the blue laser (BSSC) and the violet laser (VSSC). The set-up of the instrument is a critical point for EVs analysis, so the flow cytometer was first calibrated using the 8-Peak Rainbow Beads. The linear regression equation between the predefined fluorescence intensity values of the fluorescent microbeads and the instrument’s response in histogram channel values was then computed at several photodiode gains. The best gain was then determined for each fluorescent channel and used throughout all experiments. The Megamix-Plus FSC beads emitting a FITC-like fluorescent light of different sizes (100, 300, 500, and 900 nm), used throughout all experiments, served to establish the best photodiode gains for BSSC and VSSC, which produced the smallest coefficient of variation. Megamix-Plus FSC beads were also used for daily standardization. All signals were collected in log area mode, except the VSSC, which was also collected in log height mode and served as threshold parameter. Time delays between lasers were optimized and controlled by the standard daily QC start-up procedure.

To ensure system and reagent cleanliness, the sample line was washed with sterile distilled water filtered through 100 nm filter before each flow cytometry run, and the same fluid was used as sheath fluid. The sheath fluid tank was thoroughly rinsed with the filtered water. In between samples, the sample line was flushed by boosting filtered PBS and Coulter Clenz^®^ Cleaning Agent (Beckman Coulter, CytoFLEX S, Beckman Coulter, Milano, Italy) and clean distilled water to minimize carry-over.

Unless otherwise stated, EVs samples were run at the lowest rate allowed by the instrument (10 μL/min) to maintain the diameter of the sample stream as small as possible. Data were acquired and analysed by the CytExpert 2.2™ software (version 2.2, CytoFLEX S, Beckman Coulter, Milano, Italy). EVs number was measured using the cell-counting feature of the instrument that relies on a calibrated peristaltic pump for sample delivery.

To tighten the pulse window and thus reducing the background for small-particle analyses, the event rate setting feature implemented in the CytoFLEX S instrument was set to “high”, according to manufacturer’s instructions.

### 4.5. Western Blot Analysis

The EVs (microvesicles or exosomes) were lysed using lysis buffer (50 mmol/L Tris-HCl pH 7.2, 5 mmol/L MgCl2, 50 mmol/L NaCl, 0.25%, 0.1% SDS, and 1% Triton X-100) containing protease inhibitors (2 mmol/L phenyl methyl sulfonyl fluoride, 10 mg/mL aprotinin, and 2 mmol/L Na3VO4, 100 mmol/L NaF). Protein concentration was assessed using the Bradford method (Bradford protein assay kit II, Bio-Rad, Hercules, CA, USA), with BSA used as a standard. EVs extracted proteins (5–10 µg) were resolved by SDS PAGE (Sodium Dodecyl Sulfate PolyAcrylamide Gel Electrophoresis) 10% under reducing or non-reducing conditions, and were transferred to PVDF blotting membranes (GE Healthcare, Solingen, Germany) and analyzed using the enhanced chemiluminescence kit for Western blotting detection (Advansta, WesternBright TM ECL, Bering Drive San Jose, CA, USA). CD63 primary monoclonal antibody was used following suppliers’ (dilution, 1:500; sc-5275; Santa Cruz Biotechnology, Inc., Dallas, TX, USA). This antibody recognizes the glycosylated forms of CD63 (30–60 kDa).

### 4.6. Fluorescence Microscopy

The 50 nm anti-mouse IgG microbeads (5 μL directly from original bottle) were incubated with FITC-conjugated mouse IgG (final dilution 1:20 in filtered PBS) for 30′ at 4 °C in rotation. All images were collected with a Nikon ECLIPSE TE2000-S (Chiyoda, Tokyo, Japan) inverted microscope equipped with: 20× objective (Numerical aperture 0.4), filters TRITC, FITC, and UV, and a Nikon Digital Camera DXM1200F (Chiyoda, Tokyo, Japan). The images were analyzed with NIS Elements BR 2.10 software.

## Figures and Tables

**Table 1:** Summary of reagents.

Characteristic[s] Measured	Analyte	Analyte Detector	Reporter	Isotype	Clone	Final Concentration	Manufacturer	Catalogue. Number	Lot Number
Intracellular Esterase activity	Vesicles esterases	Calcein-green	Green-fluorescent calcein	NA	NA	1 μM	Life Technologies	C3100MP	1837717
		Calcein-violet	Violet-fluorescent calcein	NA	NA	1 μM	Life Technologies	C34858	2018203
Cell surface protein	Human CD63	Anti-human CD63 antibody	Brilliant Violet 421™	Mouse IgG1_k_	H5C6	1.25 μg/mL^−1^	BioLegend	353030	B275650
	Human CD63	Anti-human CD63 antibody	NA	Mouse IgG1_k_	H5C6	5 μg/mL^−1^	BD Pharmingen	556019	VP036
Membrane Phospholipids	Phosphatidylserine	Anti-Phosphatidylserine Antibody	Alexa Fluor 488	Mouse IgG1	1H6	1.25 μg/mL^−1^	Millipore	16-256	2926493
		Anti-Phosphatidylserine Antibody	NA	Mouse IgG1	1H6	5 μg/mL^−1^	Millipore	05-719	2867579
Mouse IgG1 Fluorescein-conjugated Antibody	Miltenyi Microbeads	50 nm-microbeads	Fluorescein	Mouse IgG1_k_	N/A	Dilution 1/20	R&D system	IC002F	1171
Anti-mouse IgG MicroBeads, human	NA	NA	NA	Mouse IgG1	NA	5 µl from the bottle	Miltenyi Biotec	130-057-501	5171201227

NA= not applicable.

## Supplementary Materials

The following are available online at https://www.mdpi.com/1422-0067/21/17/6257/s1, Figure S1: Size and morphology of isolated MVs and EXOs from HT29 cell line supernatant following differential centrifugation. Figure S2: VSSC is superior to BSSC to detect nanosized particles.
