# Supplementary material for: Measuring Extracellular Vesicles by Conventional Flow Cytometry: Dream or Reality?
Source: Int J Mol Sci. 2020 Aug 29;21(17):6257. doi: 10.3390/ijms21176257 (PMC7503575; doi:10.3390/ijms21176257)
Supplement: Supplementary file 1 [file ijms-21-06257-s001.pdf]

International Journal of *Molecular Sciences*

Supplementary Information

Cell Culture

HT29 (human colorectal adenocarcinoma) cell line was used for EVs isolation. HT29 cells were routinely propagated in Dulbecco's modified Eagle's medium, supplemented with 10% fetal bovine serum, 1% penicillin-streptomycin, and 2mmol/L L-glutamine at 37°C in a humid 5% CO2 atmosphere. Before being processed for EV isolation, HT29 cells were plated in 150 mm dishes, grown up to 70-80% of confluence and the medium replaced with serum-free medium 48 hours before collection to avoid interference by the EVs contained in the fetal bovine serum.

Dynamic Light Scattering and Transmission Electron Microscopy

Dynamic light-scattering (DLS) measurements were performed using a ZetasizerNano ZS apparatus (Malvern Instruments Ltd). Each EVs preparation is representative of five technical replicates. For morphological study, we used transmission electron microscopy; isolated microvesicles (MVs) and exosomes (EXOs) were separated as described below and fixed adding Karnovsky EM fixative (2% formaldehyde and 2.5% glutaraldehyde in 0.1 mol/L sodium cacodylate buffer, pH 7.4.) to the suspension in 1:1 ratio for 1 hour. Samples were then placed on Formvar-carbon coated grids and air dried for 10 minutes. After being rinsed with distilled water, the specimens were post fixed in 1.5% osmium tetroxide in 0.1 mol/L cacodylate buffer (pH 7.3), allowed to dry and observed with a Zeiss Libra 120 (Zeiss NTS GmbH).

EVs preparation

This study followed the protocol previously described and pre-analytical variables were tightly controlled to ensure accurate and reproducible data. Briefly, cell culture medium was centrifuged at 750*g* for 15 minutes and the supernatant centrifuged again at 1500*g* for 5 minutes. Supernatant containing the whole population of EVs, i.e., MVs and EXOs was saved and centrifuged at 17,000*g* for 45 minutes for MVs isolation. Supernatant was collected and transferred to fresh tubes to be centrifuged at 120,000*g* for 2 hours for EXOs separation. MVs and EXOs pellets were suspended in filtered (100 nm) phosphate-buffered saline (PBS) and used for the flow cytometry analysis. MV and EXO concentration was evaluated by Bradford assay. Unless otherwise stated, MV and EXO concentration was 1.5 μg/ml in all experiments. MV and EXO size was determined by Dynamic light-scattering (DLS) measurements (Fig 1a) and transmission electron microscopy (TEM) (Fig 1b) and found to be 210±49 nm and 68±7 nm (mean±DS of the five samples used for the experiments), respectively.

Flow cytometer set up

To give unobstructed view of the data, all bivariate dot plots shown in the present study display the actual cytometer outputs including the background generated by optical and electronic noise as well as by residual small particles that could not be eliminated by sheath and sample fluid filtering. All parameters are shown as signal area unless otherwise stated. The region in which the EVs were collected was pre-established by running Megamix-Plus FSC polystyrene microbeads (100 nm, 300 nm, 500 nm and 900 nm). The optimal compromise between EV analysis and background exclusion was established setting a threshold on the VSSC-height signal to include events producing a VSSC-area signal ~one and half decade lower than that generated by the smallest beads (100 nm) included in the Megamix-Plus FSC mix (Fig. 2a). Lowering the threshold further produced an excess of background signal that impeded beads recognition.

Figure 2b shows the superiority of VSSC over BSSC to resolve the Megamix-Plus FSC beads of different sizes. Only the VSSC resolved the bead aggregates that survived extensive vortexing and pipetting (pink events). VSSC also outperformed BSSC at the other measured particle sizes. The

robust CV (rCV, approximately the 75th percentile minus the 25th percentile divided by the median) of 300 nm, 500 nm and 900 nm beads was calculated through the CytExpert 2.1™ software and found to be 3.35, 4.78 and 2.93, respectively in the VSSC channel and 6.11, 5.22 and 3.75, respectively in the BSSC channel.

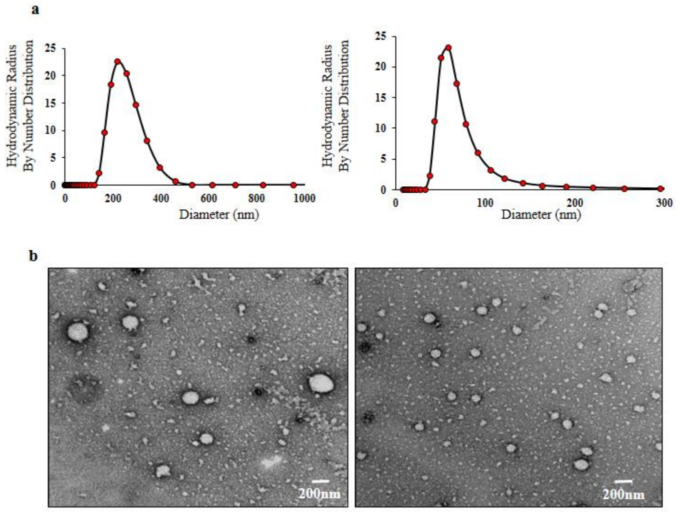

Figure 1. Size and morphology of isolated MVs and EXOs from HT29 cell line supernatant following differential centrifugation. **a**. Size distribution of MVs (left panel) and EXOs (right panel) by dynamic light scattering (DLS). **b**. Transmission electron microscopy (TEM) of MVs (left panel) and EXOs (right panel) (original magnification 49.000 ×). The experiments shown are representative for five independently performed EVs isolation experiments.

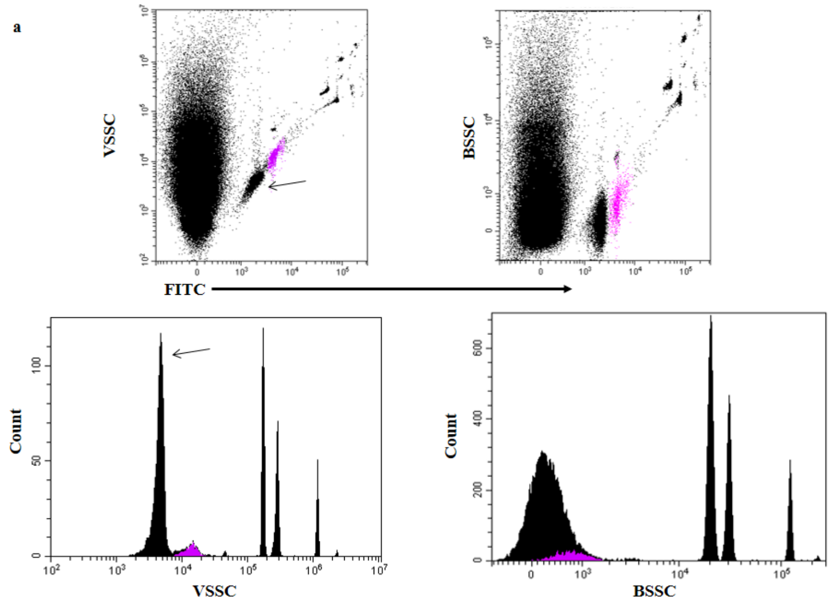

Figure 2

Figure 2. VSSC is superior to BSSC to detect nanosized particles. **a**. Dual parameter dot plots showing Megamix-Plus FSC bead (100 nm, 300 nm, 500 nm and 900 nm) data for VSSC (left panel) and BSSC (right panel). Arrow indicates the 100 nm beads. B. Single parameter plot showing the VSSC (lef panel) and BSSC (right panel) profile of the Megamix-Plus FSC beads. Arrow indicates the 100 nm beads. Note that only VSSC resolves the small amount of aggregated beads that survived vortexing (pink dots and pink curve).
